# Late Complications of Bleeding in the First 24 H After Laparoscopic Sleeve Gastrectomy: A Retrospective Cohort Study

**DOI:** 10.1007/s11695-025-08011-3

**Published:** 2025-06-23

**Authors:** Medeni Sermet, Ozgur Ekinci, Orhan Alimoglu

**Affiliations:** 1 Istanbul Medeniyet University, Istanbul, Turkey; 2 Goztepe Prof. Dr. Suleyman Yalcin City Hospital, Istanbul, Turkey

**Keywords:** Laparoscopic sleeve gastrectomy, Early postoperative bleeding, Long-term complications, Nutritional deficiencies, Muscle wasting, Sarcopenia

## Abstract

**Background:**

The objective of this study was to assess the long-term (≥ 12 months) clinical outcomes of early postoperative bleeding (within 24 h) following laparoscopic sleeve gastrectomy (LSG). The focus was on late complications such as persistent vomiting, constipation, malnutrition, gastroesophageal reflux disease (GERD), gastric stenosis, muscle wasting, and sarcopenia.

**Methods:**

A total of 463 patients who underwent LSG between 2019 and 2024 were retrospectively analyzed.Among them, 27 patients developed early postoperative bleeding, confirmed by clinical and radiologic evidence, and treated according to the Clavien-Dindo classification. All patients had at least 12 months of follow-up, during which complications were evaluated using standardized diagnostic definitions. Statistical comparisons were made between bleeding and non-bleeding groups.

**Results:**

Patients with early bleeding had significantly higher rates of persistent vomiting (33.3% vs. 6.2%), constipation (40.7% vs. 14.2%), malnutrition (29.6% vs. 6.3%), and GERD (29.6% vs. 6.2%), gastric stenosis (14.8% vs. 1.3%), and sarcopenia (mean muscle wasting: 23.7% vs. 14.8%) (*p* < 0.001). Bleeding increased the risk of severe sarcopenia (> 20% muscle wasting) by 6.2-fold.

**Conclusion:**

Bleeding within the first 24 h after LSG has a negative impact on long-term clinical outcomes, and regular follow-up and early intervention, nutritional support, and multidisciplinary follow-up strategies are critical for managing this patient group.

**Graphical Abstract:**

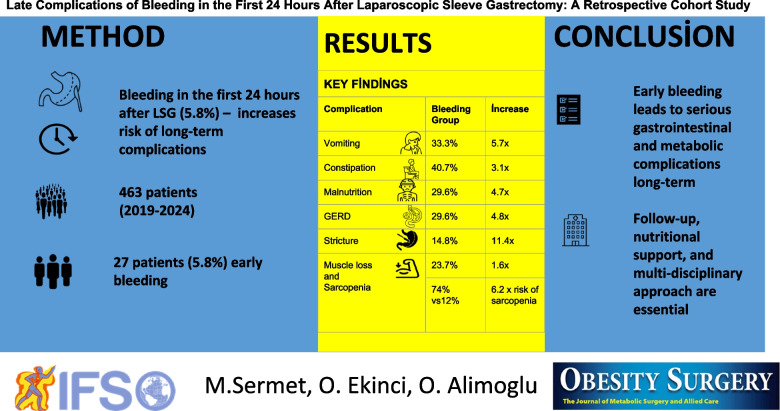

## Introduction

Bariatric surgery plays a significant role in the treatment of severe obesity, and LSG is a minimally invasive approach with a high success rate [1]. However, it is important to acknowledge that surgical complications, particularly bleeding in the early postoperative period, can potentially affect the long-term recovery process of patients. Bleeding in the early postoperative period (< 24 h) is often attributed to inadequate coagulation of microvessels in the staple line, omentum, or gastric wall [2, 3]. This can lead to hemodynamic instability, the need for transfusions, and long-term complications [4, 5].

The early consequences of these complications are known, and the main goal of early bleeding after LSG is to ensure hemodynamic stability of patients by rapid detection and intervention of bleeding complications occurring within the first 24 h after surgery. Although there are many studies in the literature on muscle wasting, nutritional deficiencies, and psychological changes after bariatric surgery, late outcomes in patients with early postoperative bleeding have not been analyzed. The potential late effects of bleeding in patients undergoing LSG may include gastroesophageal reflux, vomiting, constipation, dietary disorders, and stenosis; however, their effects on muscle wasting and sarcopenia are not yet fully understood.

This study aimed to evaluate the effects of bleeding within the first 24 h after LSG on long-term (at least 12 months) clinical outcomes, with a focus on revealing late-term outcomes such as refractory vomiting, constipation, GERD, nutritional deficiencies, gastric stricture, muscle wasting, and sarcopenia.

## Materials and Methods

### Study Design and Patient Selection

This retrospective study was based on the analysis of data from LSG performed at a university hospital bariatric surgery practice center in Turkey between 2019 and 2024. Approval for the study was obtained from the local non-interventional procedures ethics committee (2025-GOSEK-368). A total of 463 patients were included in the study, and their identification data of the included patients were anonymized, so patient consent was not required.

### Inclusion Criteria

The inclusion criteria were patients aged between 18 and 65 years, undergoing LSG only, having at least 12 months of regular clinical and laboratory follow-up, and having complete medical records.

### Exclusion Criteria

Those undergo other bariatric procedures (e.g., gastric bypass), cases requiring revision surgery, history of systemic malignancy, or severe chronic disease.

### Patient Groups

The patients were divided into two main groups:

- Group 1: Early bleeding (*n* = 27)

### Bleeding Diagnosis

Bleeding was detected clinically, radiologically, or endoscopically within the first 24 h.

Our definition of bleeding was as follows.

1. Intermittent and significant bleeding (≥ 200 mL) from intra-abdominal drainage, hematemesis, and melena causing hemodynamic impairment (hypotension, tachycardia, signs of shock).
2. Bleeding requiring one unit or more of blood transfusion.
3. Bleeding that causes a decrease in hemoglobin level ≥ 3 g/dL.
4. Bleeding that damages vital organs (e.g., intracranial, intrathoracic, intra-abdominal).
5. Postoperative bleeding requiring reoperation.

- Group 2: The control group included patients who did not develop bleeding (*n* = 436).

Our study was evaluated according to the following criteria. For all data, electronic records and examination and treatment results recorded in regular controls at 3-month intervals in accordance with the “obesity surgery practice regulation” in force in our country were used.

### Persistent Vomiting

Persistent vomiting is a complex condition. Resistant vomiting is defined as vomiting that persists for a minimum of three episodes per week for at least three months and does not respond to standard antiemetic treatments (proton pump inhibitors, prokinetic agents, and antiemetics). This definition was based on the exclusion of gastroparesis and other mechanical causes.

### Constipation

The diagnosis of constipation after LSG was defined by the presence of less than three complete stools per week, straining during defecation, or hard stools, according to the Rome IV criteria.

### Gastroesophageal Reflux Disease (GERD)

If I am not mistaken, GERD is diagnosed based on clinical symptoms (e.g., chest burning and regurgitation) and, if necessary, endoscopic evaluation [6, 7]. It is my understanding that postoperative inflammation and disturbances in gastric emptying may lead to increased reflux symptoms.

### Nutritional Deficiencies and Malnutrition

Nutritional deficiencies were evaluated based on iron, vitamin B12, folate, and protein levels. The decision was made based on the detection of low levels requiring treatment in at least one parameter as a result of laboratory tests and clinical evaluation and the detection of malnutrition in dietitian interviews.

### Stricture

The diagnostic algorithm began with upper GI contrast-enhanced radiography. For suspected luminal stenosis, we took a careful look at contrast transit time and gastric emptying pattern. When further clarity was needed, contrast-enhanced CT imaging was used as the second step. CT allowed evaluation of the stricture site’s lumen, surrounding tissues, and possible complications. In order to establish a definitive diagnosis, upper gastrointestinal endoscopy was performed. The endoscopic findings were then correlated with the results of the radiologic imaging, which allowed for the formulation of a final diagnosis.

### Muscle Loss and Sarcopenia

Muscle loss was measured using bioelectrical impedance analysis (BIA), and a ≥ 5% decrease in muscle mass after surgery was considered clinically significant. Patients with low muscle strength (hand grip test) and low muscle mass (BIA) according to the EWGSOP2 criteria for sarcopenia were selected and compared preoperatively and at 3-month intervals.

### Statistical Analysis

Statistical analysis was performed using SPSS software for Windows (version 26.0; SPSS, Inc., Chicago, IL, United States). The chi-square test was used for categorical variables and Student’s *t*-test for continuous variables, and the limit of statistical significance was set at *p* < 0.05.

## Results

### Demographic Data and Distribution of Complications

In total, 490 patients who underwent laparoscopic sleeve gastrectomy (LSG) were included in this study. Postoperative bleeding was observed in 27 patients (5.8%), while 436 patients (94.2%) completed the recovery period without such complications. Table 1 shows that the demographic data of the two groups were similar.

**Table 1:** Demographic data and additional comorbidities

Variant	Bleeding group (*n* = 27)	Non-bleeding group (*n* = 436)	*p* value
Age (mean ± SD)	37.5 ± 8.3	34.1 ± 6.1	0.28
BMI (kg/m^2^)	46.8 ± 5.2	45.9 ± 4.8	0.33
Diabetes mellitus (*n*)	10	139	0.05
Hypertension (*n*)	7	148	0.08
Dyslipidemia (*n*)	9	116	0.06
Obstructive sleep apnea (*n*)	3	83	0.12
Coronary artery disease (*n*)	1	37	0.18

*p* < 0.05 was considered significant

Patients with bleeding and interventions were classified according to the Clavien-Dindo (CD) classification, as shown in Table 2. The majority of the patients in the bleeding group was classified as having CD class 1.

**Table 2:** Patient distribution and interventions for early bleeding after LSG according to Clavien-Dindo classification

Clavien-Dindo grade	Number of patients (*n*)	Rate (%)	Treatment
Grade I	12	44.4%	Patients requiring blood transfusion
Grade II	1	3.7%	Patient undergoing bedside hematoma drainage
Grade IIIa	2	7.4%	Patients who underwent percutaneous drainage by interventional radiology for intra-abdominal abscess
Grade IIIb	3	11.1%	Patients requiring explorative surgery under general anesthesia
Grade IVa	1	3.7%	Patient with single organ failure followed in intensive care unit
Grade IVb	1	3.7%	Patient followed in intensive care unit and developed multiorgan failure
Grade V	0	0%	There have been no fatal complications

### Early Postoperative Bleeding and Long-Term Complications

Table 3 presents the findings from a statistical analysis that compared the long-term rates of adverse outcomes between patients with early bleeding and the control group.

**Table 3:** Late complications of early bleeding after LSG

Variant	Bleeding group (*n* = 27)	Non-bleeding group (*n* = 436)	*p* value
Vomiting	9 (33.3%)	27 (6.2%)	< 0.001*
Constipation	11 (40.7%)	62 (14.2%)	< 0.001*
Muscle wasting-sarcopenia (mean, %)	23.7%	14.8%	< 0.001*
Nutritional deficiency (B12, folate, iron)	8 (29.6%)	29 (6.3%)	< 0.001*
Gastric stricture	4 (14.8%)	6 (1.3%)	0.001*
GERD	8 (29.6%)	29 (6.2%)	< 0.001*

Statistical method: chi-square test was used for categorical variables and independent *t*-test for continuous variables. *p < 0.05 was considered statistically significant. *LSG* laparoscopic sleeve gastrectomy, *GERD* gastroesophageal reflux disease

The analysis revealed statistically significant differences in the long-term rates of adverse outcomes, including vomiting, constipation, muscle wasting, nutritional deficiencies, gastric stricture, and GERD, among patients with early bleeding compared with the control group.

Statistically significant differences were also found between patients with a history of early bleeding and the control group in terms of various gastrointestinal parameters.

A significant observation showed that 33.3% of patients with a history of early bleeding experienced resistant vomiting compared to only 5.8% in the control group (*p* < 0.001). This indicates that the incidence of resistant vomiting was 5.7 times higher in the hemorrhagic group than in the control group. Additionally, 40.7% of the patients with hemorrhage experienced long-term constipation versus 13.3% in the control group (*p* < 0.001), indicating that the prevalence of long-term constipation was 3.1 times higher in the hemorrhagic group.

In the hemorrhagic group, 29.6% exhibited B12, folate, and iron deficiencies compared to 6.3% in the control group (*p* < 0.001), suggesting that nutritional deficiencies were 4.7 times more prevalent in the hemorrhage group. The prevalence of gastric stricture was 14.8% in the hemorrhage group and 1.3% in the control group (*p* = 0.001), indicating that the incidence of gastric stricture increased 11.4-fold in patients with a history of hemorrhage.

**Fig. 1:**
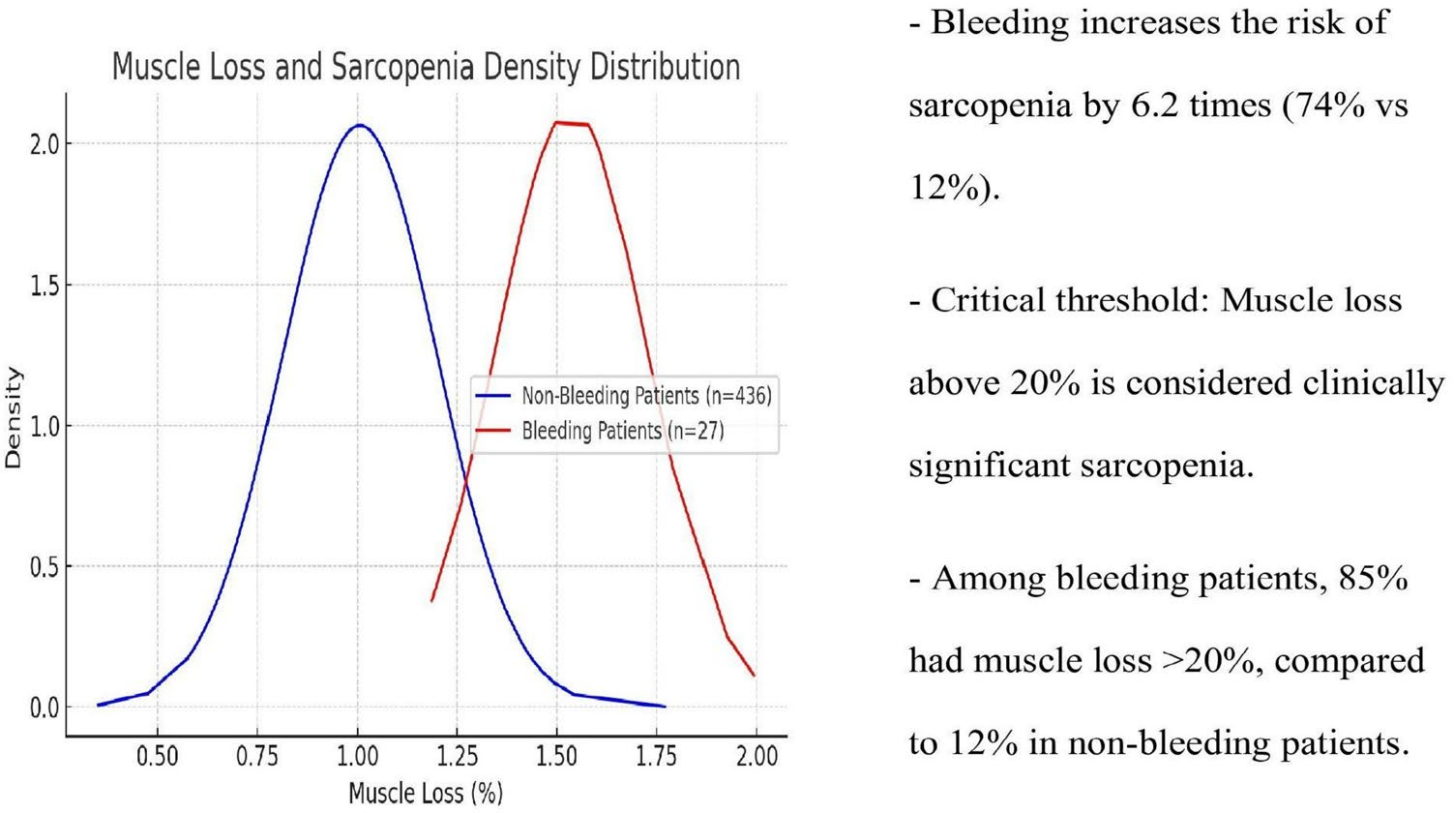
Muscle loss and sarcopenia in LSG patients with/without bleeding

The incidence of GERD was 29.6% in patients with a history of hemorrhage compared with 6.2% in the control group (*p* < 0.001), showing the GERD prevalence was 4.8 times higher in the hemorrhage group. The mean muscle wasting rate was 23.7% in the hemorrhage group and 14.8% in the control group (*p* < 0.001), indicating that the incidence of muscle atrophy incidence was 1.6 times higher in the patients with a history of hemorrhage (Table 4 and Fig. 1).

**Table 4:** Muscle loss density and sarcopenia analysis

Group	Mean loss	Standard deviation	> 20% loss ratio
Bleeding	23.7%	± 4.1%	74%
Non-bleeding	14.8%	± 3.5%	12%

All parameters showed statistically significant differences (*p* ≤ 0.001), with gastric strictures showing the most significant increase (11.4-fold). These findings suggest that patients with a history of early bleeding are at a high risk of various gastrointestinal complications in the long term.

## Dıscussıon

Early postoperative bleeding after LSG is associated with an increased incidence of long-term complications. Our study demonstrates a significant correlation between early bleeding events and the subsequent development of functional and metabolic sequelae such as vomiting, constipation, muscle wasting, and sarcopenia, nutritional deficiencies, gastric stenosis, and gastroesophageal reflux disease. These findings suggest that postoperative hemorrhagic complications extend beyond the immediate perioperative period, potentially compromising the long-term efficacy of the procedure and patient outcomes.

Our investigation revealed significant increases in vomiting (5.7-fold) and constipation (3.1-fold) in patients experiencing early postoperative bleeding. These findings suggest long-term gastrointestinal sequelae following hemorrhagic events [8]. Evidence indicates that hemorrhage-induced hypovolemia and systemic inflammatory responses may compromise vagal nerve function, resulting in gastric motility disturbances and persistent emesis [9, 10].

Concurrently, patients with postoperative hemorrhage demonstrate an elevated risk of constipation. Hemodynamic instability triggers sympathetic activation and suppresses intestinal peristalsis. Proinflammatory cytokines (IL-6, TNF-α) released following acute hemorrhage inhibit intestinal smooth muscle contractility [11, 12]. Additionally, delayed mucosal healing and reduced splanchnic perfusion extend the gastrointestinal transit time and dysfunction [13–15].

Following laparoscopic sleeve gastrectomy (LSG), early bleeding may induce anemia and persistent inflammation, impeding gastrointestinal mucosal healing and compromising absorption of iron, vitamin B12, and folate. These micronutrient deficiencies potentially limit the essential components for muscle protein synthesis, contributing to decreased muscle mass. Hemorrhage-initiated inflammation appears to disrupt the intestinal barrier integrity and exacerbate malabsorption. Research demonstrates that mucosal damage substantially reduces absorptive capacity, creating a significant obstacle to micronutrient absorption [16, 17].

Gastrointestinal mucosal damage may impair duodenal function, compromise iron carrier proteins, and potentially lead to iron deficiency anemia [16]. Persistent inflammation during early recovery may exacerbate long-term dysfunction of iron metabolism.

Vitamin B12 absorption involves a more intricate process where gastric and small intestinal mucosal damage can impair intrinsic factor production and absorption in the terminal ileum [18]. Ongoing inflammation may disrupt mucosal barrier integrity, resulting in B12 deficiency, which negatively affects erythropoiesis and neurological function [19].

The complex folate absorption process can be compromised by jejunal carrier protein dysfunction and inflammation-induced mucosal damage, contributing to folate deficiency [20]. This impairs DNA synthesis and cellular proliferation, adversely affecting metabolic balance. Chronic inflammation, oxidative stress, and elevated cytokine production further aggravate folate deficiency [21]. Additionally, postoperative alterations in the gut microbiota and intestinal barrier function may cause these issues [22].

The pathophysiological basis of systemic inflammation and mucosal damage resulting from early postoperative bleeding after LSG may precipitate severe long-term nutritional and vitamin deficiencies, potentially compromising protein synthesis and muscle mass maintenance.

Evidence suggests that early postoperative bleeding after laparoscopic sleeve gastrectomy (LSG) correlates with an increased risk of gastroesophageal reflux disease (GERD). This hemorrhagic complication negatively affects lower esophageal sphincter function through local inflammatory processes, compromised tissue healing, and delayed gastric emptying. Consequently, these pathophysiological changes facilitate retrograde flow of gastric contents, manifesting as characteristic GERD symptoms including retrosternal burning, regurgitation, and bitter oral taste.

The current literature examining post-bariatric reflux pathogenesis emphasizes the significance of inflammatory cascades and fibrotic tissue formation. Research indicates that post-surgical tissue alterations directly influence esophagogastric function, whereas ischemic injury secondary to early hemorrhage compromises gastric structural integrity, thereby promoting GERD development [23].

Therefore, longitudinal monitoring of patients with early post-LSG bleeding is imperative. Prompt identification and management of reflux symptoms may significantly enhance patients’ quality of life. Therapeutic strategies include pharmacological interventions, lifestyle modifications, endoscopic procedures, and surgical approaches.

Given the implicated role of inflammation and impaired healing in the pathophysiology of GERD, implementing multidisciplinary postoperative surveillance protocols is advisable. Vigilant monitoring of the characteristic symptoms with timely intervention is an essential component of comprehensive patient care.

Gastric stricture is a significant complication of laparoscopic sleeve gastrectomy (LSG). Evidence suggests that early postoperative bleeding may cause substantial structural alterations over time. Inflammatory processes subsequent to bariatric surgery can induce permanent gastrointestinal modifications conducive to structural changes [13]. Our investigation revealed an 11-fold increased risk of gastric stricture in patients experiencing early bleeding, potentially attributable to bleeding-induced tissue damage that triggers inflammatory cascades and compromises healing mechanisms. Local inflammation and ischemic injury following early hemorrhage may detrimentally affect gastric structural integrity, resulting in progressive luminal narrowing [24].

Clinically, strictures manifest as dysphagia, recurrent regurgitation, unexpected weight loss, and nutritional disturbances. The primary mechanisms underlying stricture development include microvascular circulation disruption, ischemic area formation, and scar tissue development [25], which are outcomes of complex interactions between inflammatory responses and tissue healing processes associated with surgical intervention.

Consequently, patients exhibiting early postoperative bleeding warrant vigilant monitoring for stricture development. Management protocols should incorporate endoscopic evaluation, dilatation procedures, and surgical revision when indicated. Long-term surveillance remains crucial for early complication detection and appropriate intervention.

Muscle wasting and sarcopenia represent potential postoperative complications. Early postoperative bleeding following LSG may correlate with complex physiopathologic processes potentially contributing to long-term muscle wasting. Hemodynamic instability and inflammatory responses triggered by early bleeding constitute primary mechanisms accelerating muscle catabolism. Elevated cortisol levels and proinflammatory cytokine release (IL-6, TNF-α) may facilitate muscle protein degradation and sarcopenia development [26–28].

Post-bleeding gastrointestinal dysmotility can induce vomiting and dysphagia, adversely affecting long-term nutrition. Difficulties transitioning to solid foods can reduce caloric and protein intake, challenging muscle mass maintenance [29, 30]. Persistent nutrient loss through vomiting negatively impacts anabolic processes, resulting in significant muscle tissue depletion [31].

Early postoperative hemorrhage following Laparoscopic Sleeve Gastrectomy (LSG) has significant implications for long-term patient outcomes. Nutritional regimen adaptation difficulties impair recovery, as delayed transition to solid foods perpetuates catabolic states, impeding muscle tissue repair [32]. Malnutrition-induced metabolic imbalances increase chronic inflammation risk [33], which can persist even after controlling early hypovolemia and inflammatory responses [34, 35].

Our study demonstrated significantly higher muscle loss rates in early postoperative hemorrhage patients (23.7%) compared to controls (14.8%) [36]. These findings suggest early bleeding negatively impacts muscle tissue, with long-term consequences including reduced physical function, metabolic irregularities, and diminished quality of life [37].

A multidisciplinary approach is crucial for this patient group, incorporating early nutritional support, regular muscle mass monitoring, inflammation parameter assessment, and physiotherapy to reduce long-term muscle loss [38].

Despite limitations of our single-center, retrospective design, this study provides valuable insights into the long-term consequences of early bleeding after LSG. Patients experiencing early postoperative bleeding face increased likelihood of late complications including vomiting, constipation, muscle wasting, nutritional deficiencies, gastric stricture, and GERD, highlighting how early bleeding complications extend beyond the immediate postoperative period, potentially impacting long-term metabolic and functional health.

## Data Availability

No datasets were generated or analysed during the current study.
